# Getting a Handle on Biosolids: New Model Estimates Microbial Exposure Risk

**Published:** 2008-06

**Authors:** Rebecca Renner

Each year several million tons of treated sewage sludge (“biosolids”) are applied to crop land, reclaimed surface mines, forests, parks, and various other land areas in the United States. Yet the public health risk from exposure to pathogens in biosolids has never been quantitatively assessed because of the lack of an appropriate model and a paucity of exposure data. Now researchers have developed a model that can estimate microbial exposure, starting with data on the content of certain pathogens in raw sewage sludge, through the treatment process, and ending with exposure to humans **[*EHP* 116:727–733; Eisenberg et al.]**.

Untreated sewage sludge contains a wide variety of microbes and parasitic worms. Current federal standards for pathogen reduction in sewage sludge are based on levels of a few indicator organisms, such as *Escherichia coli* and enteroviruses. A National Research Council committee concluded in 2002 that while there was no evidence that the standards had failed to protect public health, there also had been no concerted effort to investigate health complaints and the potential for adverse human health effects from exposure to biosolids.

In the current proof-of-concept article, the model was used to examine three pathogen exposure pathways—ingestion, drinking contaminated groundwater, and inhalation—using data for Class A biosolids, one of two EPA-designated categories of biosolids. Class A sludges have no detectible indicator organisms; low levels of indicator pathogens are permitted in class B sludges. The team used Class A biosolids data for testing.

The authors demonstrated the model’s utility by calculating human exposure in different settings. Using enterovirus concentration as a proxy for pathogens in general and beginning with data on raw sludge, they calculated the attenuation that resulted from anaerobic digestion with or without the use of lime to control the growth of pathogens. They also considered natural attenuation.

The modeling suggests that treatment systems using two anaerobic digesters substantially reduce pathogen loads. The most hazardous exposure was seen with contaminated groundwater. Ingesting a 100-mg speck of treated sludge was the next-riskiest exposure, and aerosol exposure was the least risky. Although the new model was not developed with the intention of examining specific disease outcomes, it lays the foundation for future models that could address end points such as irritation of the skin, mucous membranes, and respiratory tract.

The authors conclude that risk assessments for biosolids exposure are practical, even for Class A biosolids for which post-treatment monitoring data are below detectable limits. They also believe pathogens in biosolids can now be regulated similarly to water-related risks.

## Figures and Tables

**Figure:**
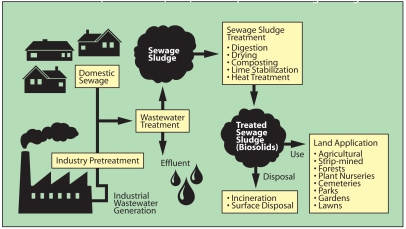
Generation, Treatment, Use, and Disposal of Sewage Sludge **Source:** Adapted from Environmental Regulations and Technology Report: Control of Pathogens and Vector Attraction in Sewage Sludge. EPA/625/R-92/013. Washington, DC: U.S. EPA, 2003: p. 1. (Matthew Ray/EHP)

